# What hinders and facilitates the implementation of nurse-led interventions in dementia care? A scoping review

**DOI:** 10.1186/s12877-020-01520-z

**Published:** 2020-04-07

**Authors:** Melanie Karrer, Julian Hirt, Adelheid Zeller, Susi Saxer

**Affiliations:** 1 Center for Dementia Care, Institute of Applied Nursing Sciences, FHS St. Gallen, University of Applied Sciences, Department of Health, Rosenbergstrasse 59, 9000 St. Gallen, Switzerland; 2 International Graduate Academy, Institute for Health and Nursing Science, Medical Faculty, Martin Luther University Halle-Wittenberg, Magdeburger Strasse 8, 06112 Halle (Saale), Germany

**Keywords:** Dementia, Implementation science, Evidence-based nursing, Barriers, Facilitators, Scoping review

## Abstract

**Background:**

The implementation of evidence-based interventions for people with dementia is complex and challenging. However, successful implementation might be a key element to ensure evidence-based practice and high quality of care. There is a need to improve implementation processes in dementia care by better understanding the arising challenges. Thus, the aim of this study was to identify recent knowledge concerning barriers and facilitators to implementing nurse-led interventions in dementia care.

**Methods:**

We performed a scoping review using the methodological framework of Arksey and O’Malley. Studies explicitly reporting on the implementation process and factors influencing the implementation of a nurse-led intervention in dementia care in all settings were included. We searched eight databases from January 2015 until January 2019. Two authors independently selected the studies. For data analysis, we used an inductive approach to build domains and categories.

**Results:**

We included 26 studies in the review and identified barriers as well as facilitators in five domains: policy (e.g. financing issues, health insurance), organisation (e.g. organisational culture and vision, resources, management support), intervention/implementation (e.g. complexity of the intervention, perceived value of the intervention), staff (e.g. knowledge, experience and skills, attitude towards the intervention), and person with dementia/family (e.g. nature and stage of dementia, response of persons with dementia and their families).

**Conclusions:**

Besides general influencing factors for implementing nursing interventions, we identified dementia-specific factors reaching beyond already known barriers and facilitators. A pre-existing person-centred culture of care as well as consistent team cultures and attitudes have a facilitating effect on implementation processes. Furthermore, there is a need for interventions that are highly flexible and sensitive to patients’ condition, needs and behaviour.

## Background

Dementia is a progressive major neurocognitive disorder characterised by impaired higher cortical functions, commonly accompanied by changes of emotional control, social behaviour, and/or motivation [1]. The increasing dependence of people with dementia leads to a need for formal or informal care [2]. In the last decades, the number of articles and the body of evidence-based nursing interventions in dementia care increased [3, 4]. However, there is a gap between the generated knowledge and its use in clinical practice. In a cross-sectional survey about research use of nurses caring for older people, only one fifth of the nurses reported the implementation of specific research findings [5]. Closing the gap between “what is known” and “what is done” is demanding. There are many challenges in transferring dementia care research into practice. In particular, the high number of recently published articles makes it difficult for clinicians to obtain an overview. A further challenge is the lack of clarity about responsibilities for translating research into practice [4]. Recent studies show that non-use or not sustainable use of evidence-based knowledge results in a lack of quality of care for people with dementia [6, 7]. Furthermore, implementing evidence-based interventions in dementia care seems complex and not sustainable. A systematic review on the effectiveness of implementation strategies in dementia care reveals that studies reporting psychosocial interventions for people with dementia rarely addressed long-term sustainability [8]. Thus, there is a need to improve implementation processes in dementia care by better understanding arising challenges. Investigating and evaluating the implementation process of interventions is crucial to figure out and to explain aspects affecting the intervention, e.g. contextual or delivery-related factors [9]. Reviews investigating challenges and influencing factors concerning the implementation of interventions in dementia care are focused on psychosocial interventions in residential dementia care or on personalised dementia care in community settings [8, 10]. They describe intervention-related, personal, financial and organisational characteristics, management support as well as the willingness of residents and/or families to participate as influencing factors in the implementation process. In a scoping review addressing implementation strategies as well as related barriers and facilitators in dementia care, Lourida et al. [11] identified factors influencing implementation and dissemination activities in dementia care from publications between 1998 and October 2015. They describe the following categories: organisational (e.g. time, workload, leadership) and professional (e.g. knowledge and training), personality and staff characteristics (e.g. engagement, resistance), financial (e.g. funding), environmental (physical structure), legal, resident-specific (health status) and dementia-specific (cognitive impairment and complications in the course of the disease). The authors showed that research activities in this area rapidly increased over time. One third of the studies were published in 2014 and 2015 [11]. This indicates the importance of investigating the most recently published literature regarding factors hindering and facilitating implementation processes in this field.

## Methods

The aim of our study was to identify the recent knowledge concerning barriers and facilitators to implementing nurse-led interventions in dementia care. We conducted a scoping review following the methodological framework of Arksey and O’Malley [12]: (1) identifying the research question, (2) identifying relevant studies, (3) selecting studies, (4) charting the data, (5) collating, summarizing and reporting the results. This type of review is described as a form of knowledge synthesis mapping key concepts, types of evidence and research gaps [13]. We used PRISMA-ScR for reporting [14]. An internal review protocol guided the process.

### Identifying the research question and determining criteria for inclusion and exclusion

To answer our research question “What hinders and facilitates the implementation of nurse-led interventions in dementia care?”, we included peer reviewed studies with a qualitative, quantitative or mixed-methods design in English or German. Studies should address care for people with dementia (all types and stages) or dyads consisting of people with dementia and their relatives. We included studies investigating the implementation process, i.e. factors influencing the implementation of a nurse-led intervention (e.g. barriers, facilitators, difficulties, enablers, challenges). We defined nurse-led interventions as interventions predominately performed by nurses. The population of interest (i.e. persons asked about barriers and facilitators) consisted of people with all types and stages of dementia, relatives of people with dementia and health professionals involved in the implementation process. Thus, participants asked about barriers and facilitators were not necessarily those who performed the intervention. We included all settings (e.g. long-term care, acute care hospitals, outpatient settings). We excluded studies investigating the effectiveness of the intervention (without formal process evaluation examining the implementation process). Furthermore, we did not include studies focusing on compliance with an intervention, adherence to an intervention or acceptance of an intervention. We excluded studies presenting interventions mainly conducted by physicians or pharmacists, interventions focusing only on relatives of people with dementia as well as diagnostic or preventive interventions. Since Lourida et al. [11] conducted their literature search until October 2015 we limited our search to the period from January 2015 to January 2019.

### Identifying relevant studies

We conducted a comprehensive literature search comprising (i) eight databases (CINAHL, MEDLINE via Ovid, Emcare, PsycINFO via Ovid, Embase via Ovid, CENTRAL via Cochrane Library, Web of Science Core Collection, Ovid Nursing Database), (ii) handsearching of relevant journals not indexed in the chosen databases (journal/ISSN: Angewandte Gerontologie Appliquée/2297–5179, Pflegezeitschrift/2520–1816, Klinische Pflegeforschung/2365–7863, QuPuG/2414–6889), (iii) free web searching via Google Scholar as well as (iii) backward and forward citation tracking of included studies using Scopus. If not indexed in Scopus, we manually searched reference lists and performed forward citation tracking by means of Google Scholar.

MK designed the search strategy using elements of Lourida et al. [11] with kind permission of the corresponding author. She further identified search terms based on existing topic-specific literature by means of an orientating search via different databases. JH reviewed the search strategy using PRESS [15]. We used controlled vocabulary as well as free search terms to circumvent the issue of delayed indexing of controlled vocabulary [16]. The search string included two components. One component contained search terms for dementia and Alzheimer’s disease, the second component comprised search terms for implementation processes, e.g. “program implementation”, “diffusion of innovation”, “barriers and facilitators” or “knowledge to action”. We used the following search techniques: Boolean and proximity operators as well as wildcards. Additional file 1 shows our final search strategies for each database.

### Selecting studies

Independently, MK and JH systematically checked titles, abstracts, and full texts regarding inclusion and exclusion criteria. Discrepancies were resolved by discussion.

### Charting the data

For extraction, MK and JH elaborated a standardised data charting form including the following information: country, aim, setting, study design, implemented intervention, participants, data collection, data analysis, and main results. MK extracted the data, JH and SAX checked randomly chosen 50 % for reasons of accuracy. Since no data extraction errors were identified, we decided not to check the second half of the studies.

### Collating and summarizing the results

We used an inductive approach for data analysis [17]. First, MK tagged all barriers and facilitators mentioned throughout the results sections of included studies. Afterwards, MK and JH coded all tags in accordance with the passage in the text. MK then created categories out of the codes. To increase the trustworthiness of the data analysis concerning accuracy, credibility and transferability, SAX and AZ peer-checked the resulting system of categories. Finally, we discussed the system of categories and adapted it in collaboration with all authors. We used MAXQDA 2018 for data analysis.

## Results

**Fig. 1:**
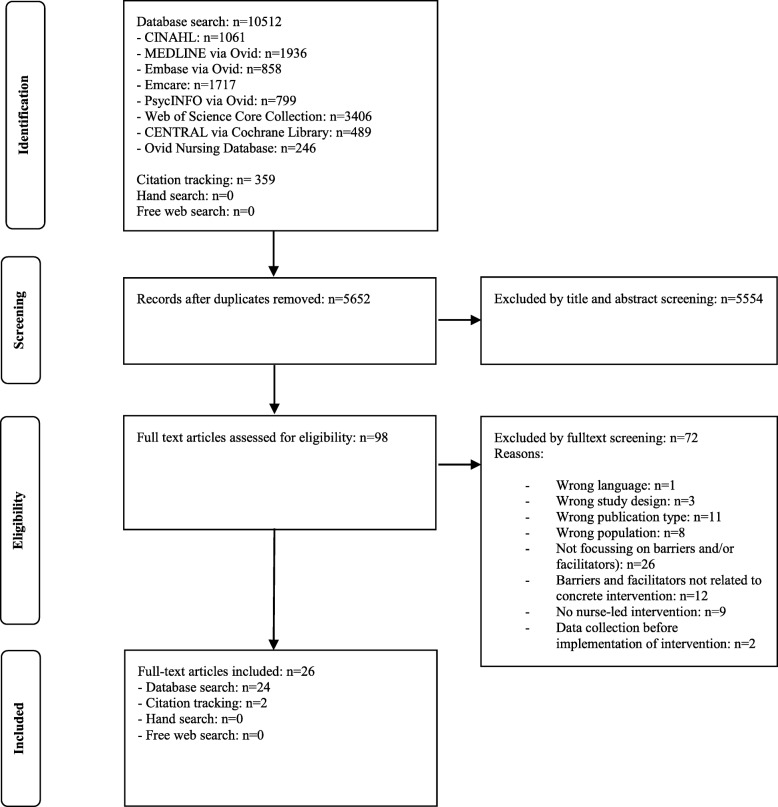
Flow chart of the search and selection process

The search in January 2019 yielded a total of 5652 references after removal of duplicates. Of these, we included 98 publications for full text screening. We excluded 72 publications for the following reasons: wrong language (*n* = 1), wrong study design (*n* = 3), wrong publication type (*n* = 11), wrong population (*n* = 8), not focussing on barriers and/or facilitators (*n* = 26), barriers and facilitators not related to a concrete intervention (*n* = 12), no nurse-led intervention (*n* = 9), data collection before implementing the intervention and impossibility to describe actually experienced barriers and facilitators (*n* = 2). Finally, we included 26 studies in our review. Fig. 1 shows the search and selection process in detail.

### Characteristics of included studies

Table 1 displays characteristics of included studies. Most of the studies were from the Netherlands [19, 21, 29, 30, 37, 42] and the UK [23, 25, 28, 32, 33, 40] (each *n* = 6), followed by Australia [24, 34, 41], Canada [20, 22, 27], Norway [26, 31, 36] (each *n* = 3), Germany [38, 39], and Belgium [18, 43] (each *n* = 2). One multinational study took place in Italy and the Netherlands [35]. All articles were written in English. 80% of the studies were published since 2017 [18–22, 24, 26–28, 30–37, 39–41, 43] (*n* = 21). Most of the studies were conducted in the long-term care setting [18, 19, 21–28, 30–39, 42, 43] (*n* = 22), two were performed in an acute hospital [40, 41] and one in the outpatient setting [20]. One study had a mixed setting (outpatient and long-term care) [29]. Four studies used the “Promoting Action on Research Implementation in Health Services” (PARiHS) framework as a theoretical framework for implementation [20, 26, 31, 36]. One study was based on the normalisation process theory [32]. The remaining publications used a conceptual framework for implementation of advance care planning [43] (*n* = 1) and the “COM-B (capability, opportunity and motivation-behaviour) system” [41] (*n* = 1) as a framework. Nineteen studies did not refer to a specific implementation framework.

**Table 1:** Characteristics of included studies

Number (referring to Table 2) Author (year) Country Setting Study design^1^	Intervention description Theoretical framework for implementation	Participants
1. Ampe et al. (2017) [18] Belgium Nursing Home (Dementia Care wards) CCT	Multidisciplinary communication intervention “we DECide” for nursing home staff in order to ensure shared decision making in advance care planning conversations with residents affected by dementia and their families.	Multidisciplinary nursing home healthcare teams on the management and the clinical level (*n* = 90)
2. Appelhof et al. (2018) [19] Netherlands Nursing Home (Special Care units for People with young-onset dementia) RCT	Intervention based on the “Grip on Challenging Behavior” care program aiming to improve the management of NPS in persons with young-onset dementia	Nurses, psychologist, physicians and team leaders (*n* = 82)
3. Bayly et al. (2018) [20] Canada Outpatient Setting Multiple case study	Implementation of dementia-focused “integrated Knowledge Transfer” strategies by a “knowledge broker” (nurse) to facilitate knowledge transfer between health care professionals and people with dementia and their relatives. PARiHS Framework	Rural home care providers: Registered and licensed practical nurses, health care aides, managers, and other care providers (*n* = 19)
4. Boersma et al. (2017) [21] Netherlands Nursing Home (Psycho-geriatric wards) Multiple case study	The “Veder Contact Method” combining core components of existing psychosocial and person-centred methods in dementia care in order to improve the contact between caregivers and people with dementia.	Professional caregivers and managers involved in the study (*n* = 54)
5. Bourbonnais et al. (2018) [22] Canada Nursing Home Action research study	A complex intervention developed to manage screaming in older people with dementia. Theories on changing practice and building new habits	Formal (registered nurses, licensed practical nurses, nurse aides, special education instructors, managers) caregivers (*n* = 16) and family caregivers (*n* = 3)
6. Brooker et al. (2016) [23] UK Nursing Home Mixed-methods study	The “Focussed Intervention Training and Support” programme to reduce antipsychotic prescribing for people with dementia.	Dementia care coaches and university-based educators designated Dementia Practice Development Coaches (*n* = 68)
7. Chenoweth et al. (2018) [24] Australia Nursing Home Before and after study	Multifaceted intervention to support antipsychotic deprescribing for people with dementia.	Champions of the intervention (senior registered nurses, clinical nurse specialist, clinical nurse consultant, nurse practitioner, quality managers, deputy director of nursing, care unit managers) (*n* = 22)
8. Clark et al. (2016) [25] UK Nursing Home Not clear	“Sporting memories work” to engage older people with dementia.	Leaders and staff involved in the study (*n* = not indicated)
9. Dahl et al. (2018) [26] Norway Nursing Home c-RCT	A tailored educational intervention focused on reducing relational and physical restraint for people with dementia. PARiHS Framework	Nursing home staff (*n* = NI)
10. Ducak et al. (2018) [27] Canada Nursing Home Qualitative study	“Montessori Methods for Dementia” using a person-centred approach to increase participation in, and enjoyment of, daily life of people with dementia.	Nursing home staff in the recreation/programs/activities department, managers/educators or regulated health care professional (*n* = 17)
11. Griffiths et al. (2019) [28] UK Nursing Home RCT	DCM aimed to allow care home staff delivering more person-centred care for people with dementia.	Care home managers, DCM mappers, staff members, expert mappers (*n* = 75), residents (*n* = 2) and relatives (*n* = 6)
12. Hendriks et al. (2016) [29] Netherlands Different settings (meeting and day care centres, long-term care institutions) Qualitative study	Personalized nature activities to support well-being and quality of life of people with dementia.	Professionals (*n* = 13), volunteers (*n* = 3) and people with dementia (*n* = 12) involved in the intervention
13. Henskens et al. (2017) [30] Netherlands Nursing Home CCT	“Movement-oriented restorative care” to optimize independence in activities of daily living and quality of life of people with dementia.	Nurses, activity supervisors, heads of department, physiotherapist, occupational therapist, ‘ambassadors’ (*n* = 12)
14. Jacobsen et al. (2017) [31] Norway Nursing Home Mixed-Methods study	Educational intervention to support shared decision-making to avoid the use of restraint in agitated residents with dementia. PARIHS Framework	Quantitative data: nursing home staff (*n* = 452) Qualitative data: Nurses, auxiliary nurses, nursing assistants, social educators, occupational therapists (*n* = 53)
15. Keenan et al. (2018) [32] UK Nursing Home c-RCT including case studies	E-learning and decision support intervention to support nursing home staff in interacting with residents displaying challenging behaviours. Normalisation Process Theory	Home managers, care staff, research intervention nurse and therapist (*n* = 9)
16. Latham et al. (2017) [33] UK Nursing Home Mixed-methods study and case studies	The “Focussed Intervention Training and Support” programme to reduce inappropriate antipsychotic prescribing for people with dementia.	Dementia care coaches, staff, managers (*n* = 30)
17. Luckett et al. (2017) [34] Australia Nursing Home RCT	Facilitated case conferencing with family decision-makers in order to improve quality of end of life care in nursing home residents with advanced dementia.	Registered Nurses in the PCPC role, other members of nursing home staff, and physicians participating in case conferences (*n* = 40)
18. Mariani et al. (2017) [35] Italy and Netherlands Nursing Home Qualitative study	Multicomponent intervention to improve shared decision-making.	Healthcare professionals (mostly healthcare assistants) involved in the study (n = 19)
19. Mekki et al. (2017) [36] Norway Nursing Home C-RCT	Educational intervention to support shared decisions to avoid the use of restraint in agitated residents with dementia. PARIHS Framework	Facilitators of the intervention (n = 8)
20. Pieper et al. (2018) [37] Netherlands Nursing Home Mixed-methods Study	“STA OP!” multicomponent intervention to reduce symptoms of pain and challenging behaviour in people with dementia.	Healthcare professionals participating in the intervention (n = 6)
21. Quasdorf et al. (2016) [38] Germany Nursing Home CCT	DCM to enhance person-centred care.	Head nurses, staff nurses, project coordinators (*n* = 27)
22. Quasdorf et al. (2019) [39] Germany Nursing Home Case study	DCM to enhance person-centred care.	Head nurses, staff nurses, project coordinators (*n* = 28)
23. Surr et al. (2018) [40] UK Acute Hospital Case study	Training interventions to improve practice and care experiences for people with dementia.	Dementia training facilitators and staff having attended training, ward managers (*n* = 49)
24. Toye et al. (2019) [41] Australia Acute Hospital Mixed-methods study	A systematic nurse–caregiver conversation to provide safe person-centred hospital care for people with dementia. COM-B system (capability, opportunity and motivational/behavioural system)	Nurses (*n* = 6)
25. Van Mierlo et al. (2015) [42] Netherlands Nursing Home Qualitative study	Mental health care transfer intervention after admission to a nursing home of a person with dementia in order to promote continuity of care.	Community psychiatric nurses, professional home carers, stakeholders (*n* = 27) and family caregivers (*n* = 5)
26. Wils et al. (2017) [43] Belgium Nursing Home Before and after study	Educational program for nursing staff to improve advanced care planning. Conceptual framework for implementation of advance care planning	Nurses (*n* = 13)

^1^Study design of the overall study (e.g. of the implementation or evaluation study, where barriers and facilitators were investigated in an embedded sub-study or independent qualitative studies)

Abbreviations: *CCT* Controlled clinical trial, *c-RCT* Cluster-RCT, *DCM* Dementia Care Mapping, *NI* No information available, *NPS* Neuropsychiatric symptoms, *PARiHS* Promoting Action on Research Implementation in Health Services, *RCT* Randomized controlled trial

Data concerning barriers and facilitators were collected through interviews [10, 20–41, 43] (*n* = 24), by means of questionnaires [19, 23, 24, 31, 38] (*n* = 6), field notes or process data notes [26, 32, 37, 38] (*n* = 4), observation [25, 39, 40] (*n* = 3), workshops [36] (*n* = 1), written evaluations by trainers/instructors [37] (*n* = 1), residents’ records [38] (*n* = 1) and/or by asking open-ended questions [18] (*n* = 1). Qualitative data were analysed using thematic or content analysis [18–27, 29, 31–42] (*n* = 23) or framework analysis [28, 32] (*n* = 2). Quantitative data analysis was based on multilevel regression analysis [31] (*n* = 1) or descriptive statistics [38] (*n* = 1). Participants were mostly health professionals on different hierarchical levels (e.g. registered nurses, healthcare assistants), from different disciplines (e.g. nurses, physicians, psychologists) and working on management or clinical level. All were part of the intervention or the implementation (*n* = 23 studies). Family caregivers were asked in three studies [22, 28, 42] and people with dementia in two studies [28, 29]. Three studies included persons facilitating the intervention [24, 30, 36, 40] and one study included volunteers [29]. The number of participants ranged between six and 90. All studies, except for one [41], reported barriers and facilitators.

### Barriers and facilitators

We identified five domains of barriers and facilitators: policy, organisation, intervention/implementation, staff and person with dementia/family. Within these domains, we created one to twelve categories describing influencing factors (barriers or facilitators) (Table 2). For full information about extracted data, see additional file 2: Summary of included studies.

**Table 2:** Domains and Categories of identified barriers and facilitators

	Barriers		Facilitators	
DOMAIN	Categories	References	Categories	References
Policy	Financing issues	25	Health insurance organisation	25
	Governmental regulations	10		
Organisation	Organisational culture and vision	1,2,4,5, 7,10, 11,15,16,18,21,22,25	Organisational culture and vision	3,4,7,15,16,18,21,22
	Management and leader support and commitment	4,11,14,15,16,19,20,21,23	Management and leader support and commitment	1,2,3,4,5,7,9,10,11,14,15,17,19,20,21,22, 23,26
	Resources	1,2,3,4,5,6,8,9,10,11,12,13,15,16,17,18,20,23,24,25,26	Resources	1,2,4,10,11,12,13,15,17,18,25
	Staff turnover and fluctuation	2,5,15,17,20,22,23,25		
	Demands competing with the intervention	2,4,5,8,9,11,16,17,20, 21,23		
Intervention/ Implementation	Perceived value of the intervention	9,15,19,20,25	Perceived value of the intervention	4,8,10,11,17,20
	Sufficiency of intervention training delivery	4,23	Sufficiency of intervention training delivery	1,4,6,8,10,11,13,16,18,19,23
	Degree of clarity of the intervention	4,11,15,21	Degree of clarity of the intervention	1,4,5,7,11,15,17,20,21
	Suitability for current practice	2,15,20	Suitability for current practice	3,4,20
	Environmental conditions	12	Environmental conditions	12
	Support from defined persons	11,22	Support from defined persons	2,3,6,7,10,11,14,15,16
	Qualification and enthusiasm of the supplying person	11,15,16,23	Qualification and enthusiasm of the supplying person	15,16,23
	Conditions for the supplying person	11,16,19	Conditions for the supplying person	6,11,16
	Collaboration with stakeholders	16,25	Involvement of staff in intervention development and delivery	3,19
	Implementation methods	3,6,19	Involvement of multiple disciplines and hierarchical levels	1,2,3,4,11,13,16,18,20,21,22,25
	Complexity of the intervention	2,3,4,7,11,12,15,18,25		
	Issues concerning the trial procedure	6,7,15,19		
Staff	Team cultures	4,5,20,22,25	Team cultures	1,3,4,6,7,8,10,12,17,19,22,23,25
	Knowledge, experience and skills of staff	1,4,8,11,12,19,22,23,25	Knowledge, experience and skills of staff	1,11,12,18,19
	Motivation and energy of staff	4,9,12,19,22,23	Motivation and openness of staff	2,4,5,11,12,15,18,19,22,23,25
	Degree and clarity of responsibilities	1,4,21,25		
	Degree of familiarity with the intervention	4,5,12,18,25		
	Attitude towards the intervention	4,5,6,7,11,15,17,22,23		
	Focus of care	4,9		
Person with Dementia/Family	Family engagement	4,5,18,23	Family engagement	5,10,16,18
	Attitude towards the intervention on the part of the family and other patients	7,23	Response to the intervention on the part of persons with dementia and the family	4,10,17
	Nature and stage of dementia	3,4,9,12,13,18,26	Education, knowledge and experience of the person with dementia and the family	10,12,18
	Background information about the person with dementia	4		

The references in this table do not refer to the references in the text. They refer to the numbers given in Table 1

#### Policy

The policy domain describes enabling or hindering factors on the governmental or municipal level.

Barriers: *Financing issues*, e.g. no clear reimbursement for the delivery of an intervention [42] or *governmental regulations* concerning task-oriented practices [27] are considered as barriers in the implementation process.

Facilitators: The authors of one study [42] mention the organization of *health insurance* promoting collaboration of dementia care networks and allowing reimbursement of intervention delivery as a facilitating factor.

#### Organisation

We categorised factors relating to characteristics, structures and processes of an organisation (e.g. nursing home, hospital) in the organisation domain.

Barriers: The category *organisational culture and vision* summarizes influencing factors. Distinctive hierarchical structures [18, 27, 28, 36, 38, 39], inadequate regulations within the organisation [21, 35] and a task-focused, functional culture of care [34, 38, 39] are examples for hindering factors within this category. Furthermore, authors of several studies report a lack of *management and leader support and engagement* as a hindering factor [21, 28, 31–33, 36–38, 40]. This was the case when leaders were passive or authoritative or when their role was not clear. Limited *resources*, e.g. lack of financial support, staff, time, space and material, are described as barriers [18–23, 25–30, 32–35, 37, 40–43].

High rates of *staff turnover and fluctuation* [19, 22, 32, 34, 37, 38, 40, 42] as well as *demands competing with the intervention* [19, 21, 22, 25, 26, 28, 33, 34, 37, 39, 40] are mentioned as further barriers. *Competing demands* comprise other innovations or organisational changes taking place at the same time as well as multiple and contradictory requirements.

Facilitators: Some authors describe a facilitating *organisational culture and vision* if a person-centred culture was already established [38, 39] and the hierarchies were flat [20, 24, 38, 39].

*Committed and supportive leaders and managers* are mentioned as facilitators [18–22, 24, 26–28, 31, 32, 34, 36–40, 43]. This also applies to adequate *resources* (staff, time, budget, space) [18, 19, 21, 27–30, 32, 34, 35, 42].

#### Intervention and implementation

In the intervention and implementation domain, we identified facilitating and hindering factors concerning characteristics of intervention content and delivery as well as features of the implementation process.

Barriers: Within the category *perceived value of the intervention* a barrier is described if the impact or effect of the intervention is not obvious to staff [26, 32, 36, 37, 42]. Another category mentioned in the studies is *sufficiency of intervention training delivery*. This is considered a barrier if training was not sufficient, e.g. with regard to staff participation or due to scheduling outside regular working hours [21, 40]. In the category *degree of intervention clarity,* uncertainties concerning the intervention, or the implementation are mentioned as hindering factors [21, 28, 32, 38]. Furthermore, authors describe it as hindering if the intervention was not *suitable for current practice,* e.g. if it overlapped with current working methods [19, 32, 37]. Hendriks et al. [29] mention *environmental conditions* and describe bad weather as a barrier to outdoor intervention. The category *support from a defined person* proves to be hindering if a defined and sensitive person is missing [28, 38]. Thus, lacking *qualification and enthusiasm of the supplying person* [28, 32, 33, 40] are considered as barriers. This is also the case regarding *conditions for the supplying person,* e.g. concerning the use of personal resources, overload due to organisational requirements or missing support from staff [28, 33, 36].

C*ollaboration with stakeholders* is described as a barrier if relevant stakeholders did not want to be involved in the intervention [33, 42]. Concerning the category *implementation methods,* unfamiliar methods, e.g. teleconference supervisions or online communication can hinder the implementation process [20, 23, 36]. We also identified the *complexity of the intervention* as a barrier. Authors of integrated studies describe time-consuming, complex and expensive interventions as hindering successful implementation [19–21, 24, 28, 29, 32, 35, 42]. Barriers summarized in the category *issues concerning trial procedure* comprise the organisation of the trial, follow-up or supervision periods and communication problems between the study team and staff in clinical practice [23, 24, 32, 36].

Facilitators: We recognized *experience of the value of an intervention* as a facilitator. Seeing the positive results of an intervention, e.g. enhanced quality of care and quality of life of the person with dementia, is motivating for staff [21, 25, 27, 28, 34, 37]. Furthermore, *sufficiency of intervention training delivery* can influence the implementation processes. Authors of various studies describe this category as facilitating if the training followed a practical or interactive approach [21, 35, 40]. Additionally, some authors mention the *degree of clarity of the intervention* as an influencing factor. They report clear and structured interventions as facilitators [18, 21, 22, 24, 28, 32, 34, 37, 43]. The category *suitability for current practice* covers enabling factors like applicability of an intervention to daily practice [21] as well as alignment with current organisational structures and procedures [20, 37]. Furthermore, several authors describe *support from a defined person* as an enabling factor [19, 20, 23, 24, 27, 28, 31–33]. If the supplying person is *qualified and enthusiastic* [32, 33, 40] and the *conditions for the supplying person* [23, 28, 33] are good, this proved to be facilitating. Examples for good conditions are support from clinical staff and from another supplying person, sufficient time and respect on the part of the organisation. *Involvement of staff in intervention development and delivery* [20, 36] as well as *involvement of multiple disciplines and hierarchical levels* are described as facilitators in various studies [18–21, 28, 30, 33, 35, 37–39, 42].

#### Staff

Barriers or facilitators within the staff domain refer to factors directly related to staff characteristics, qualities and attitudes.

Barriers: It became obvious that certain characteristics of *team cultures,* e.g. different cultures within a team, inefficient communication, inflexible team members or conflicts within the team can impede the implementation [21, 22, 37, 38, 42]. Moreover, *staff knowledge, experience and skills* can influence implementation processes. Insufficient dementia-specific or intervention-specific knowledge or missing confidence regarding dementia care are described as barriers [18, 21, 25, 28, 29, 38, 40, 42]. Various authors mention a lack of *staff motivation and energy* as a barrier [21, 26, 29, 36, 38, 40]. If staff is not committed or displays passive behaviour during the implementation process, this is described as hindering. *Limited or unclear responsibilities*, e.g. non-transparent intervention-related responsibilities can negatively influence staff motivation and thus adversely affect the implementation process [18, 21, 38, 42]. *The degree of familiarity with the intervention* also seems to be an influencing factor. Some authors describe it as hindering, if staff is not familiar with the intervention or feels uncomfortable with it [21, 22, 29, 35, 42]. In this context, *staff attitude towards the intervention* is relevant as well. Negative attitudes, e.g. scepticism, resistance or lack of acceptance regarding the intervention are identified as barriers [21–24, 28, 32, 34, 38, 40]. Furthermore, the *focus of care* on to-do task lists and the accompanying prioritisation of efficiency hinders the implementation of nurse-led interventions in dementia care [21, 26].

Facilitators: Positive *team cultures* ensuring mutual support and well-functioning collaboration and communication are described as facilitators [18, 20, 21, 23–25, 27, 29, 34, 36, 38, 40, 42]. Furthermore, *staff knowledge, skills and experience* can positively influence the implementation if staff is highly qualified and confident [18, 28, 29, 35, 36]. *Staff motivation and openness* are also mentioned as influencing factors. Various authors describe motivated and enthusiastic staff with high commitment to change as a beneficial factor [8, 10, 19, 22, 28, 29, 32, 35, 36, 39, 40].

#### Person with dementia and family

In this domain, enabling and hindering factors refer to characteristics of the recipients of an intervention, i.e. people with dementia and their relatives.

Barriers: Lacking *engagement of families* can complicate the implementation of a nurse-led intervention in dementia care. If relatives are not available or if they are not willing to engage in the intervention process, this is mentioned as hindering [21, 22, 35, 40]. Moreover, negative *attitudes towards the intervention on the part of family caregivers or other patients* are described as barriers [24, 40].

The category *nature and stage of dementia* includes hindering factors directly relating to characteristics of the person with dementia. Residents’ or patients’ cognition, particularly quickly changing needs and fluctuating behaviours of people with dementia are challenging in the implementation process [20, 21, 26, 29, 30, 35, 43]. Furthermore, in one study, lack of *background information about the person with dementia* is mentioned as a barrier [21].

Facilitators: The *engagement of families* is described as an important factor. Several authors mention strong relationships with relatives and involvement of relatives as supportive [22, 27, 33, 35]. Positive *response of people with dementia and their families*, e.g. positive feedback or cheerful reactions are also described as facilitating factors [21, 27, 34]. Moreover*, education, knowledge and experience of the person with dementia and the family* are mentioned as enabling factors as well [27, 29, 35].

## Discussion

In our scoping review on barriers and facilitators of implementation processes in dementia care, we identified influencing factors in five domains: policy, organisation, intervention/implementation, staff and person with dementia/family. In the following, we will discuss our results in the context of Lourida et al. [11] who reviewed studies published until October 2015. We identified dementia-specific factors reaching beyond the already known general barriers and facilitators to implementing nursing interventions with other patient groups. Overall, our results are consistent with the influencing factors described by Lourida et al. [11]. Moreover, we identified additional barriers and facilitators mentioned in most recent publications. In the organisational domain, Lourida et al. [11] describe time constraints, increased workload, leadership and managerial support as influencing factors. Furthermore, our results show that organisational culture and vision are essential for implementing processes in dementia care. This is mentioned in over 50 % of the studies. Distinctive hierarchical structures, inadequate regulations within the organisation [21, 35], and a task-focused, functional culture of care [24, 38, 39] are described as barriers. In contrast, a person-centred culture of care [38, 39] and flat hierarchies are perceived as enabling factors [20, 24, 38, 39].

A pre-existing person-centred culture of care in the organisation seems to be a factor of particular importance. Thus, implementing nursing interventions in dementia care is deemed to be more successful in organisations with already established person-centred care principles as components of a care philosophy and an organisation of care. In a systematic review addressing barriers and facilitators to general implementation processes in hospitals, Geerligs et al. described the culture of the system as an influencing factor in combination with staff workload, lack of time, and high staff turnover. The culture of the system comprises the attitudes of employees as well as organisational readiness to change [44]. This is far beyond the culture of care as such. In contrast, Vlaeyen et al. [45] did not mention culture as a facilitating factor at all in their systematic review on barriers and facilitators to implementing fall prevention in residential care facilities. Staffing, training, and the interest of the organisation proved to be more important [45].

The types of interventions addressed in our review give further insight into the reasons why a person-centred culture of care might be important for success. The included studies reported, for example, on communication-based and psychosocial and interventions as well as on interventions related to behavioural and psychological symptoms of dementia. As the included publications show, interventions in the context of dementia are often complex and consist of multicomponent interventions with a person-centred approach.

In a person-centred culture of care, staff attitudes and qualifications seem to be an important influencing factor. Lourida et al. [11] mention professional factors, for example dementia-related knowledge, tool-based training as well as staff experience and personality. The degree of commitment to change or resistance proves to be an influencing factor as well. This is in line with our findings concerning the staff domain, for example “staff knowledge, experience and skills” as well as “staff motivation and openness”. We further identified team cultures, attitudes towards the intervention and familiarity with the intervention as influencing factors. In 50% of the studies, authors described team cultures mainly as a facilitating factor. This was the case if team members experienced collaboration and communication as motivating and supportive. In this context, each professional’s individual focus of care was mentioned as influencing, e.g. with regard to a task-oriented or a person-oriented dementia care.

Literature reviews addressing other patient groups confirm that staff knowledge, skills and attitudes are important factors in the implementation process [44, 45]. Our results show additional dementia-specific factors: professional’s individual focus of care and conflicts due to different cultures of care within the team. Task-oriented or efficiency-oriented staff attitudes were described as hindering factors. Regarding other interventions, e.g. emergency interventions of fall prevention programs, an efficiency-oriented approach would not be hindering to that extent and as well could be a facilitator.

Moreover, we identified person-related and family-related barriers as well as facilitators in line with the results of Lourida et al. [11]. In the studies they analysed, poor health status and cognitive impairment were reported only once. In our review, factors directly relating to characteristics of the person with dementia were described in more than one quarter of included studies [20, 21, 26, 29, 30, 35, 43]. Publications with other patient groups also reported on patient- or resident-related influencing factors. Attitude towards the intervention and physical and mental disability [46] as well as motivational and compliance-related aspects were mentioned [45]. Our results highlight the difficulty of implementing pre-defined interventions with respect to the fluctuating needs and behaviours of people with dementia. This is a highly dementia-specific factor requiring well-considered development of interventions.

Regarding the transfer of results into practice, it should be considered that barriers and facilitators described in the included studies are predominately reported from health professional’s perspective. Only in four studies, relatives were involved and people with dementia participated only in two studies. Thus, further process evaluations in this field should consider persons with dementia and their families to a greater extent. Including people with dementia in research is considered as essential in order to ensure a benefit for future patients [47]. By excluding people with dementia and their families, highly relevant dementia-specific influencing factors could be overseen. Moreover, future studies should focus more detailed on the dementia-specific factors described in this review.

With 80% of included studies published since 2017, our scoping review confirms the trend towards an increasing number of research investigating implementation processes in dementia care as mentioned by Lourida et al. [11]. Thus, ongoing consideration of published articles in this field is necessary.

### Strengths and limitations

A strength of our study is the comprehensive database search as well as the systematic approach to study selection and data analysis. By means of an additional free web search and backward as well as forward citation tracking, we tried to identify all relevant studies in this field. However, we probably have overseen relevant studies, particularly due to heterogenous publication venues. Data extraction by only one author is a methodological limitation. Only half of the studies were checked by another author. Furthermore, a more precise differentiation of some categories was not possible since some categories refer to different domains. For example, staff attitudes and staff focus of care are part of the organisational culture and value. On the other hand, they are separate factors as well. Nevertheless, our results provide an overview of crucial factors influencing the implementation of interventions in dementia care.

## Conclusion

This scoping review contributes to the knowledge about implementation processes in dementia care. It can serve as a basis to inform future implementation of dementia-specific interventions. Thus, it can contribute to close the gap between generated knowledge on interventions and its use in clinical practice. Notably, to ensure successful interventions for persons with dementia, systems supporting a person-centred culture of care are required, e.g. a person-centred approach to leadership. Furthermore, a consistent understanding of dementia care within the team as well as communication and collaboration are crucial factors for implementing interventions in dementia care. Given the complexity of the care situation, successful interventions need to be flexible and sensitive to patients’ current condition, needs and behaviours.

## Supplementary information

**Additional file 1.** Database-specific search strategies.

**Additional file 2.** Summary of included studies.

## Data Availability

All data generated or analysed during this study are included in this published article and its supplementary information files.

## Abbreviations

CCT: Controlled clinical trial
COM-B: Capability, opportunity and motivation-behaviour
c-RCT: Cluster-RCT
DCM: Dementia care mapping
NI: No information available
NPS: Neuropsychiatric symptoms
PARiHS: Promoting action on research implementation in health services
PRESS: Peer review of electronic search strategies
RCT: Randomized controlled trial

## References

[1] World Health Organization (2016). International Statistical Classification of Diseases and Related Health Problems 10^th^ Revision.

[2] Alzheimer’s Disease International. World Alzheimer Report 2013 - Journey of Caring: An analysis of long-term care for dementia. London; 2013. https://www.alz.co.uk/research/WorldAlzheimerReport2013.pdf.

[3] Gitlin LN, Marx K, Stanley IH, Hodgson N (2015). Translating evidence-based dementia caregiving interventions into practice: state-of-the-science and next steps. Gerontologist..

[4] Draper B, Low L-F, Withall A, Vickland V, Ward T (2009). Translating dementia research into practice. Int Psychogeriatr..

[5] Boström A-M, Kajermo KN, Nordström G, Wallin L (2009). Registered nurses’ use of research findings in the care of older people. J Clin Nurs..

[6] Souza R, Gandesha A, Hood C, Chaplin R, Young J, Crome P, Crawford MJ (2014). Quality of care for people with dementia in general hospitals: national cross-sectional audit of patient assessment. Clin Med..

[7] Bökberg C, Ahlström G, Karlsson S. Significance of quality of care for quality of life in persons with dementia at risk of nursing home admission: a cross-sectional study. BMC Nurs. 2017;16(39) 10.1186/s12912-017-0230-6. 10.1186/s12912-017-0230-6 PMC5513341 28725160

[8] Boersma P, van Weert JCM, Lakerveld J, Dröes RM (2015). The art of successful implementation of psychosocial interventions in residential dementia care: a systematic review of the literature based on the RE-AIM framework. Int Psychogeriatr..

[9] Moore GF, Audrey S, Barker M, Bond L, Bonell C, Hardeman W (2015). Process evaluation of complex interventions: Medical Research Council Guidance. BMJ..

[10] van Mierlo LD, Meiland FJM, van Hout HPJ, Dröes RM (2016). Toward an evidence-based implementation model and checklist for personalized dementia care in the community. Int Psychogeriatr..

[11] Lourida I, Abbott RA, Rogers M, Lang IA, Stein K, Kent B, Thompson CJ (2017). Dissemination and implementation research in dementia care: a systematic scoping review and evidence map. BMC Geriatr..

[12] Arksey H, O’Malley L (2005). Scoping studies: towards a methodological framework. Int J Soc Res Methodol..

[13] Colquhoun HL, Levac D, O’Brien KK, Straus S, Tricco AC, Perrier L (2014). Scoping reviews: time for clarity in definition, methods, and reporting. J Clin Epidemiol..

[14] Tricco AC, Lillie E, Zarin W, O’Brien KK, Colquhoun HL, Levac D (2018). PRISMA extension for scoping reviews (PRISMA-ScR): checklist and explanation. Ann Intern Med..

[15] McGowan J, Sampson M, Salzwedel DM, Cogo E, Foerster V, Lefebvre C (2016). PRESS peer review of electronic search strategies: 2015 guideline statement. J Clin Epidemiol..

[16] Irwin AN, Rackham D (2017). Comparison of the time-to-indexing in PubMed between biomedical journals according to impact factor, discipline, and focus. Res Soc Adm Pharm..

[17] Schreier M (2012). Qualitative content analysis in practice.

[18] Ampe S, Sevenants A, Smets T, Declercq A, van Audenhove C (2017). Advance care planning for nursing home residents with dementia: influence of ‘we DECide’ on policy and practice. Patient Educ Couns..

[19] Appelhof B, Bakker C, van Duinen-van den IJssel JCL, Zwijsen SA, Smalbrugge M, Verhey FRJ (2018). Process evaluation of an intervention for the management of neuropsychiatric symptoms in young-onset dementia. J Am Med Dir Assoc.

[20] Bayly M, Forbes D, Blake C, Peacock S, Morgan D (2018). Development and implementation of dementia-related integrated knowledge translation strategies in rural home care. Online J Rural Nurs Health Care..

[21] Boersma P, van Weert JC, van Meijel B, Droes RM (2017). Implementation of the Veder contact method in daily nursing home care for people with dementia: a process analysis according to the RE-AIM framework. J Clin Nurs..

[22] Bourbonnais A, Ducharme F, Landreville P, Michaud C, Gauthier M-A, Lavallee M-H. An Action Research to Optimize the Well-Being of Older People in Nursing Homes: Challenges and Strategies for Implementing a Complex Intervention. J Appl Gerontol. 2018; Article in Press. 10.1177/0733464818762068. 10.1177/0733464818762068 29504489

[23] Brooker DJ, Latham I, Evans SC, Jacobson N, Perry W, Bray J (2016). FITS into practice: translating research into practice in reducing the use of anti-psychotic medication for people with dementia living in care homes. Aging Ment Health..

[24] Chenoweth L, Jessop T, Harrison F, Cations M, Cook J, Brodaty H (2018). Critical contextual elements in facilitating and achieving success with a person-Centred care intervention to support antipsychotic Deprescribing for older people in long-term care. Biomed Res Int..

[25] Clark M, Murphy C, Jameson-Allen T, Wilkins C (2017). Sporting memories, dementia care and training staff in care homes. J Ment Health Train Educ Pract.

[26] Dahl H, Dewing J, Mekki TE, Håland A, Øye C (2018). Facilitation of a workplace learning intervention in a fluctuating context: an ethnographic, participatory research project in a nursing home in Norway. Int Pract Dev J..

[27] Ducak K, Denton M, Elliot G (2018). Implementing Montessori methods for dementia ™ in Ontario long-term care homes: recreation staff and multidisciplinary consultants’ perceptions of policy and practice issues. DEMENTIA..

[28] Griffiths AW, Kelley R, Garrod L, Perfect D, Robinson O, Shoesmith E (2019). Barriers and facilitators to implementing dementia care mapping in care homes: results from the DCM™ EPIC trial process evaluation. BMC Geriatr..

[29] Hendriks IH, van Vliet D, Gerritsen DL, Droes RM (2016). Nature and dementia: development of a person-centered approach. Int Psychogeriatr..

[30] Henskens M, Nauta IM, Scherder EJA, Oosterveld FGJ, Vrijkotte S (2017). Implementation and effects of movement-oriented restorative care in a nursing home - a quasi-experimental study. BMC Geriatr..

[31] Jacobsen FF, Mekki TE, Forland O, Folkestad B, Kirkevold O, Skar R (2017). A mixed method study of an education intervention to reduce use of restraint and implement person-centered dementia care in nursing homes. BMC Nurs..

[32] Keenan J, Poland F, Manthorpe J, Hart C, Moniz-Cook E. Implementing e-learning and e-tools for care home staff supporting residents with dementia and challenging behaviour: A process evaluation of the ResCare study using normalisation process theory. Dementia. 2018; Article in Press. 10.1177/1471301218803195. 10.1177/1471301218803195 PMC7309360 30269534

[33] Latham I, Brooker D (2017). Reducing anti-psychotic prescribing for care home residents with dementia. Nurse Prescribing..

[34] Luckett T, Chenoweth L, Phillips J, Brooks D, Cook J, Mitchell G (2017). A facilitated approach to family case conferencing for people with advanced dementia living in nursing homes: Perceptions of palliative care planning coordinators and other health professionals in the IDEAL study. Int Psychogeriatr..

[35] Mariani E, Vernooij-Dassen M, Koopmans R, Engels Y, Chattat R (2017). Shared decision-making in dementia care planning: barriers and facilitators in two European countries. Aging Ment Health..

[36] Mekki TE, Øye C, Kristensen B, Dahl H, Haaland A, Nordin KA (2017). The inter-play between facilitation and context in the promoting action on research implementation in health services framework: a qualitative exploratory implementation study embedded in a cluster randomized controlled trial to reduce restraint in nursing homes. J Adv Nurs..

[37] Pieper MJC, Achterberg WP, Van Der Steen, Jenny T, Francke AL (2018). Implementation of a Stepwise, Multidisciplinary Intervention for Pain and Challenging Behaviour in Dementia (STA OP!): A Process Evaluation. Int J Integr Care.

[38] Quasdorf T, Riesner C, Dichter MN, Dortmann O, Bartholomeyczik S, Halek S (2016). Implementing dementia care mapping to develop person-centred care: results of a process evaluation within the Leben-QD II trial. J Clin Nurs..

[39] Quasdorf T, Bartholomeyczik S (2019). Influence of leadership on implementing dementia care mapping: a multiple case study. Dementia..

[40] Surr CA, Sass C, Burnley N, Drury M, Smith SJ, Parveen S, et al. Components of impactful dementia training for general hospital staff: A collective case study. Aging Ment Health. 2018; Article in Press. 10.1080/13607863.2018.1531382. 10.1080/13607863.2018.1531382 30596270

[41] Toye C, Slatyer S, Quested E, Bronson M, Hill A, Fountaine J, et al. Obtaining information from family caregivers to inform hospital care for people with dementia: A pilot study. Int J People Nurs. 2019; Article in Press. 10.1111/opn.12219. 10.1111/opn.12219 30628766

[42] van Mierlo LD, Bootsma-Van der Wiel A, Meiland FJM, van Hout HPJ, Stek ML, Droes RM (2015). Tailored mental health care after nursing home admission: improving transfers of people with dementia with behavioral problems. An explorative study. Aging Ment Health..

[43] Wils M, Verbakel J, Lisaerde J (2017). Improving advance care planning in patients with dementia: the effect of training nurses to engage in ACP-related conversations. J Clin Gerontol Geriatr..

[44] Geerligs L, Rankin NM, Shepherd HL, Butow P (2018). Hospital-based interventions: a systematic review of staff-reported barriers and facilitators to implementation processes. Implement Sci..

[45] Vlaeyen E, Stas J, Leysens G, van der Elst E, Janssens E, Dejaeger E (2017). Implementation of fall prevention in residential care facilities: a systematic review of barriers and facilitators. Int J Nurs Stud..

[46] Korall AMB, Feldman F, Scott VJ, Wasdell M, Gillan R, Ross D (2015). Facilitators of and barriers to hip protector acceptance and adherence in long-term care facilities: a systematic review. J Am Med Dir Assoc..

[47] Thorogood A, Mäki-Petäjä-Leinonen A, Brodaty H, Dalpé G, Gastmans C, Gauthier S (2018). Consent recommendations for research and international data sharing involving persons with dementia. Alzheimers Dement..

