# Supplementary material for: What hinders and facilitates the implementation of nurse-led interventions in dementia care? A scoping review
Source: BMC Geriatr. 2020 Apr 7;20:127. doi: 10.1186/s12877-020-01520-z (PMC7140366; doi:10.1186/s12877-020-01520-z)
Supplement: Supplementary file 2 — Additional file 2. Summary of included studies. [file 12877_2020_1520_MOESM2_ESM.docx]

**Additional file 2: Summary of included studies**

| Author (year)  Country  Setting  Study design | Study design^^[[1]](#footnote-1)^^  Intervention description | Review specific study characteristics | | | Review specific findings - Categories | |
| --- | --- | --- | --- | --- | --- | --- |
|  |  | **Aim** | **Participants** | **Data collection**  **Data analysis** | **Barriers** | **Facilitators** |
| Ampe et al. (2017)  Belgium  Nursing Home (Dementia Care wards) | CCT  The communication intervention “we DECide” for nursing home staff for realizing shared decision making in advanced care planning conversations with residents with dementia and their families. | To investigate barriers and facilitators for implementing “we DECide”. | Nursing home staff from different disciplines of the management and the clinical level (n=90) | Data collection: Asking two open-ended questions  Data analysis: Thematic analysis | - Organisational culture and vision - Resources - Knowledge, experience and skills of staff - Degree and clarity of responsibilities | - Management and leader support and engagement - Resources - Sufficiency of intervention training delivery - Degree of clarity of the intervention - Involvement of multiple disciplines and hierarchical levels - Team cultures - Knowledge, experience and skills of staff |
| Appelhof et al. (2018)  Netherlands  Nursing Home (Special Care units for People with young-onset dementia) | RCT  Intervention based on the “Grip on Challenging Behavior” care program which is aimed to improve the management of neuropsychiatric symptoms in young-onset dementia. It contains the systematic detection and a structured analysis of neuropsychiatric symptoms and development of a treatment plan with a specific goal. | To provide information about the implementation strategy and factors affecting implementation. | Nurses, psychologist, physicians and team leaders (n=82) | Data collection: Open-ended questions on a questionnaire  Data analysis: Content analysis | - Organisational culture and vision - Resources - Demands competing with the intervention - Staff turnover and fluctuations - Suitability to current practice - Complexity of the intervention | - Management and leader support and engagement - Resources - Support through defined persons - Involvement of multiple disciplines and hierarchical levels - Motivation and openness of staff |
| Bayly et al. (2018)  Canada  Outpatient Setting | Multiple case study  Implementation of dementia-focused “integrated Knowledge Transfer” strategies through a knowledge broker (nurse) to facilitate the knowledge transfer between health care professionals and people with dementia and their relatives. | To examine the process  and contextual factors which facilitate or hinder the development and implementation of dementia care “integrated Knowledge Transfer” strategies. | Rural home care providers: Registered and licensed practical nurses, health care aides, managers, and other care providers (n=19) | Data collection: Semi-structured interviews  Data analysis: Thematic analysis | - Resources - Implementation methods - Complexity of the intervention - Nature and stage of dementia | - Organisational culture and vision - Management and leader support and engagement - Suitability to current practice - Support through defined persons - Involvement of staff in intervention development and delivery - Involvement of multiple disciplines and hierarchical levels - Team cultures |
| Boersma et al. (2017)  Netherlands  Nursing Home (Psycho-geriatric wards) | Multiple case study  The “Veder Contact Method” which combines core components from existing psychosocial and person-centred methods in dementia care, to improve the contact between caregivers and people with dementia. such as reminiscence, validation, integrated emotion-oriented care and neuro-linguistic programming, with the use of elements like theatrical, musical and poetic communication. | To perform a process analysis of the implementation of the  “Veder Contact Method” for gaining insight into factors that influence successful  Implementation. | Professional caregivers and managers involved in the study (n=54) | Data collection: Focus group interviews, semi-structured interviews  Data analysis: Thematic analysis | - Organisational culture and vision - Management and leader support and engagement - Resources - Demands competing with the intervention - Sufficiency of intervention training delivery - Degree of clarity of the intervention - Complexity of the intervention - Team cultures - Knowledge, experience and skills of staff - Motivation and energy of staff - Degree and clarity of responsibilities - Degree of familiarity with the intervention - Attitude towards intervention - Focus of care - Engagement of families - Nature and stage of dementia - Background information on the person with dementia | - Organisational culture and vision - Management and leader support and engagement - Resources - Perceived value of the intervention - Sufficiency of intervention training delivery - Degree of clarity of the intervention - Suitability to current practice - Involvement of multiple disciplines and hierarchical levels |
| Bourbonnais et al. (2018)  Canada  Nursing Home | Action Research Study  A complex intervention developed to manage the screams of older people living with dementia. | To assess strategies useful in implementing a complex intervention based on the meanings of screams of older people living in Nursing homes with Alzheimer’s disease and related disorders. | Formal (registered nurses, licensed practical nurses, nurses’ aides, special education instructors, managers) caregivers (n=16) and family caregivers (n=3) | Data collection: Focus group interviews, semi-structured interviews  Data analysis: Content analysis | - Organisational culture and vision - Resources - Staff turnover and fluctuations - Demands competing with the intervention - Team cultures - Degree of familiarity with the intervention - Engagement of families | - Management and leader support and engagement - Degree of clarity of the intervention - Motivation and openness of staff - Engagement of families |
| Brooker et al. (2016)  UK  Nursing Home | Mixed-methods Study  The “Focussed Intervention Training and Support” programme to reduce antipsychotic prescribing for people with dementia. | To explore the experience of implementing the “Focussed Intervention Training and Support” programme. | Dementia care coaches and University-based educators designated Dementia Practice Development Coaches (n=68) | Data collection: Open-ended questionnaire, semi-structured interviews  Data analysis: Thematic analysis | - Resources - Implementation methods - Issues concerning the trial procedure - Attitude towards intervention | - Sufficiency of intervention training delivery - Support through defined persons - Conditions for the supplying person - Team cultures |
| Chenoweth et al. (2018)  Australia  Nursing Home | Before and after study  Multifaceted intervention to support antipsychotic deprescribing for people with dementia using a train-the trainer approach to deliver person-centred dementia care education and support, dementia management education, awareness raising and use of an individualised deprescribing protocol. | To identify the champions’  experiences with applying the person-centred care training when providing leadership to direct care staff in person-centred dementia care. | Champions of the intervention (senior registered nurses, clinical nurse specialist, clinical nurse consultant, nurse practitioner, quality managers, deputy director of nursing, care unit managers) (n=22) | Data collection: Open-ended questionnaire, semi-structured interviews  Data analysis: Content analysis | - Organisational culture and vision - Complexity of the intervention - Issues concerning the trial procedure - Attitude towards intervention - Attitudes towards intervention from family and other patients | - Organisational culture and vision - Management and leader support and engagement - Degree of clarity of the intervention - Support through defined persons - Team cultures |
| Clark et al. (2016)  UK  Nursing Home | Not clear  “Sporting memories work”: uses individual and collective memories of sport as a basis for reminiscence work to engage older people with dementia. Staff had a half-day sports memories training and got support materials and support from Sporting memories staff over telephone or e-mail. | To develop a better understanding of how to implement sporting reminiscences in residential care, particularly for people with dementia and those with depressed mood. | Lead persons and staff involved in the study (n= not clear) | Data collection: Interviews, observation, survey  Data analysis: Thematic analysis | - Resources - Demands competing with the intervention - Knowledge, experience and skills of staff | - Perceived value of the intervention - Sufficiency of intervention training delivery - Team cultures |
| Dahl et al. (2018)  Norway  Nursing Home | c-RCT  A tailored education intervention focused on reducing relational and physical restraint for people with dementia. | To investigate what local contextual circumstances influenced the process of  facilitation of the intervention. | Nursing home staff (n= NI) | Data collection: Interviews, field notes  Data analysis: Three-part analysis (hermeneutic knowledge co-production, ethnographic thematic analysis, general thematic analysis) | - Resources - Demands competing with the intervention - Perceived value of the intervention - Motivation and energy of staff - Focus of care - Nature and stage of dementia | - Management and leader support and engagement |
| Ducak et al. (2018)  Canada  Nursing Home | Qualitative Study  The “Montessori Methods for Dementia” uses a person-centred approach to increase participation in, and enjoyment of, daily life of people with dementia. | To investigate recreation staff and multidisciplinary consultants’ perceptions  of factors that affect the implementation of Montessori Methods for Dementia in long-term care homes. | Nursing home staff worked in the recreation/programs/activities department, managers/educators or regulated health care professional (n=17) | Data collection: Telephone interviews  Data analysis: Thematic analysis | - Financing issues - Ministry regulations - Organisational culture and vision - Resources | - Management and leader support and engagement - Sufficiency of intervention training delivery - Support through defined persons - Team cultures - Engagement of families - Reactions of people with dementia and families to intervention - Education, knowledge and experience of person with dementia and family |
| Griffiths et al. (2019)  UK  Nursing Home | RCT  DCM aimed to help care home staff deliver more person-centred care for people with dementia. Two staff members from each intervention care home were trained to use DCM and then asked to implement three DCM cycles, each comprising of briefing; observation; data analysis, reporting and feedback; and action planning. | To report the barriers and facilitators to DCM implementation. | Care home managers, DCM mappers, staff members, expert mappers (n=75), residents (n=2) and relatives (n=6) | Data collection: Semi-structured interviews  Data analysis: Framework analysis approach | - Organisational culture and vision - Management and leader support and engagement - Resources - Demands competing with the intervention - Degree of clarity of the intervention - Support through defined persons - Qualification and enthusiasm of supplying person - Conditions for the supplying person - Complexity of the intervention - Knowledge, experience and skills of staff - Attitude towards intervention | - Management and leader support and engagement - Resources - Perceived value of the intervention - Sufficiency of intervention training delivery - Degree of clarity of the intervention - Support through defined persons - Conditions for the supplying person - Involvement of multiple disciplines and hierarchical levels - Knowledge, experience and skills of staff - Motivation and openness of staff |
| Hendriks et al. (2016)  Netherlands  Different settings (meeting and day care centres, long-term care institutions) | Qualitative Study  Personalized nature activities to support well-being and quality of life of people with dementia. such as nature walk, gardening, and sensory activation in nature) that fitted their preferences best. The activities were offered in a group context. | To investigate, if personalized nature activities for persons with dementia are feasible according to professionals in care practice. | Professionals (n=13), volunteers (n=3) and people with dementia (n=12) who were involved in the intervention | Data collection: Semi-structured interviews  Data analysis: Thematic analysis | - Resources - Environmental conditions - Complexity of the intervention - Knowledge, experience and skills of staff - Motivation and energy of staff - Degree of familiarity with the intervention - Nature and stage of dementia | - Resources - Environmental conditions - Team cultures - Knowledge, experience and skills of staff - Motivation and openness of staff - Education, knowledge and experience of person with dementia and family |
| Henskens et al. (2017)  Netherlands  Nursing Home | CCT  “Movement-oriented restorative care” to optimize independence in activities of daily living and quality of life of people with dementia. | To administer process evaluations to determine the extent to which “Movement-oriented restorative care” was implemented as intended. | Nurses, activity supervisors, heads of the departments, physiotherapist, occupational therapist, ‘ambassadors’ (n=12) | Data collection: Focus group interviews  Data analysis: NI | - Resources - Nature and stage of dementia | - Resources - Sufficiency of intervention training delivery - Involvement of multiple disciplines and hierarchical levels |
| Jacobsen et al. (2017)  Norway  Nursing Home | Mixed-Methods Study  Education intervention to support shared decisions to avoid the use of restraint in agitated residents with dementia. The facilitators provided coaching sessions to assist the staff in applying the decision-making model. | To investigate which factors hindered or facilitated staff awareness related to confidence building initiatives based on person-centred care. | Quantitative Data: Nursing home staff (n= 452)  Qualitative Data: Nurses, nursing auxiliaries, assistant nurses, social educators, occupation therapists (n=53) | Quantitative Data:  Data collection: Person-centred Care Assessment Tool, The General Nordic questionnaire for psychological and social factors at work  Data analysis: Multilevel regression analysis  Qualitative Data:  Data collection: Interviews  Data analysis: Three stages (thematic and context specific thematic coding procedure, focused coding procedure, analysis with the PARIHS framework) | - Management and leader support and engagement | - Management and leader support and engagement - Support through defined persons |
| Keenan et al. (2018)  UK  Nursing Home | c-RCT including Case Studies  An e-learning and decision support intervention to help care home staff support residents with challenging behaviours. | To report on the contextual and organisational mechanisms, barriers  and facilitators. | Home managers, care staff, research intervention nurse and therapist (n=9) | Data collection: Interviews, notes  Data analysis: Framework analysis, thematic analysis | - Organisational culture and vision - Management and leader support and engagement - Resources - Staff turnover and fluctuations - Perceived value of the intervention - Degree of clarity of the intervention - Suitability to current practice - Qualification and enthusiasm of supplying person - Complexity of the intervention - Issues concerning the trial procedure - Attitude towards intervention | - Organisational culture and vision - Management and leader support and engagement - Resources - Degree of clarity of the intervention - Support through defined persons - Qualification and enthusiasm of supplying person - Motivation and openness of staff |
| Latham et al. (2017)  UK  Nursing Home | Mixed-methods Design and Case studies  The “Focussed Intervention Training and Support” programme to reduce inappropriate antipsychotic prescribing for people with dementia. | To elucidate facilitators and barriers to successful implementation of the FITS into practice approach. | Dementia care coaches, staff, managers (n= 30) | Data collection: Interviews  Data analysis: Thematic analysis | - Organisational culture and vision - Management and leader support and engagement - Resources - Demands competing with the intervention - Qualification and enthusiasm of supplying person - Conditions for the supplying person - Collaboration with stakeholders | - Organisational culture and vision - Sufficiency of intervention training delivery - Support through defined persons - Qualification and enthusiasm of supplying person - Conditions for the supplying person - Involvement of multiple disciplines and hierarchical levels - Engagement of families |
| Luckett et al. (2017)  Australia  Nursing Home | RCT  Facilitated case conferencing with family decision-makers for improving the quality of end of life care in nursing home residents with advanced dementia. | To explore Palliative Care Planning Coordinators and health professional perceptions of the benefits of facilitated case conferencing and identify factors influencing implementation. | Registered Nurses in the PCPC role, other members of nursing home staff, and physicians who participated in case conferences (n=40) | Data collection: Semi-structured interviews  Data analysis: Thematic analysis | - Resources - Staff turnover and fluctuations - Demands competing with the intervention - Attitude towards intervention | - Management and leader support and engagement - Resources - Perceived value of the intervention - Degree of clarity of the intervention - Team cultures - Reactions of people with dementia and families to intervention |
| Mariani et al. (2017)  Italy and Netherlands  Nursing Home | Qualitative Study  Multicomponent intervention to improve shared decision making. First, professionals involved in care planning and residents’ care had to attend a communication skills training course; second, they had to perform shared decision-making interviews with residents with dementia and their family caregivers to identify and prioritize needs; third, residents’ life-and-care plans were tailored to their actual needs and preferences. | To identify barriers and facilitators regarding the implementation of a shared decision-making framework for care planning. | Healthcare professionals (mostly healthcare assistants) involved in the study (n=19) | Data collection: Focus group interviews  Data analysis: Content analysis | - Organisational culture and vision - Complexity of the intervention - Degree of familiarity with the intervention - Engagement of families - Nature and stage of dementia | - Organisational culture and vision - Resources - Sufficiency of intervention training delivery - Involvement of multiple disciplines and hierarchical levels - Knowledge, experience and skills of staff - Motivation and openness of staff - Engagement of families - Education, knowledge and experience of person with dementia and family |
| Mekki et al. (2017)  Norway  Nursing Home | c-RCT  Education intervention to support shared decisions to avoid the use of restraint in agitated residents with dementia. The facilitators provided coaching sessions to assist the staff in applying the decision-making model. | To explore the inter-play between external facilitation and nursing home contexts relative to intervention outcomes. | Facilitators of the intervention (n=8) | Data collection: Focus group interviews and workshops  Data analysis: Content analysis | - Management and leader support and engagement - Perceived value of the intervention - Conditions for the supplying person - Implementation methods - Issues concerning the trial procedure - Knowledge, experience and skills of staff - Motivation and energy of staff | - Management and leader support and engagement - Sufficiency of intervention training delivery - Involvement of staff in intervention development and delivery - Team cultures - Knowledge, experience and skills of staff - Motivation and openness of staff |
| Pieper et al. (2018)  Netherlands  Nursing Home | Mixed-methods Study  “STA OP!” multicomponent intervention to reduce symptoms of pain and challenging behaviour of people with dementia. | To examine the extent to which the “STA OP!” intervention was delivered and implemented as intended and to understand factors influencing the implementation process. | Healthcare professionals who participated in the intervention (n=6) | Data collection: Semi-structured interviews, written evaluations, notes and memos  Data analysis: Thematic Analysis | - Management and leader support and engagement - Resources - Demands competing with the intervention - Staff turnover and fluctuations - Perceived value of the intervention - Suitability to current practice - Team cultures | - Management and leader support and engagement - Perceived value of the intervention - Degree of clarity of the intervention - Suitability to current practice - Involvement of multiple disciplines and hierarchical levels |
| Quasdorf et al. (2019)  Germany  Nursing Home | Case Study  DCM to enhance person-centred care. | To investigate how the leaders of the participating Nursing Homes influenced the implementation of DCM. | Head nurses, staff nurses, project coordinators (n=28) | Data collection: Semi-structured interviews  Data analysis : Content analysis | - Organisational culture and vision - Management and leader support and engagement - Demands competing with the intervention - Degree of clarity of the intervention - Degree and clarity of responsibilities | - Organisational culture and vision - Management and leader support and engagement - Degree of clarity of the intervention - Involvement of multiple disciplines and hierarchical levels |
| Quasdorf et al. (2016)  Germany  Nursing Home | CCT  DCM to enhance person-centred care. | To describe what factors facilitate and hinder DCM implementation. | Head nurses, staff nurses, project coordinators (n=27) | Data collection: Semi-structured interviews, Dementia Institution Questionnaire, Dementia Milieu Assessment, resident records, process documents  Data analysis: Content analysis, descriptive statistics | - Organisational culture and vision - Staff turnover and fluctuations - Support through defined persons - Team cultures - Knowledge, experience and skills of staff - Motivation and energy of staff - Attitude towards intervention | - Organisational culture and vision - Management and leader support and engagement - Involvement of multiple disciplines and hierarchical levels - Team cultures - Motivation and openness of staff |
| Surr et al. (2018)  UK  Acute Hospital | Case Study  Training interventions to improve practice and better care experiences for people  with dementia. | To investigate what were the specific barriers and facilitators to  effective training implementation for healthcare staff. | Dementia training lead, staff who facilitated delivery of the training, staff who had attended training, ward managers (n=49) | Data collection: Focus group interviews, small group Interviews, individual interviews, DCM  Data analysis: Thematic analysis | - Management and leader support and engagement - Resources - Staff turnover and fluctuations - Demands competing with the intervention - Sufficiency of intervention training delivery - Qualification and enthusiasm of supplying person - Knowledge, experience and skills of staff - Motivation and energy of staff - Attitude towards intervention - Engagement of families - Attitudes towards intervention from family and other patients | - Management and leader support and engagement - Sufficiency of intervention training delivery - Qualification and enthusiasm of supplying person - Team cultures - Motivation and openness of staff |
| Toye et al. (2019)  Australia  Acute Hospital | Mixed-methods Study  A systematic nurse–caregiver conversation intervention to provide safe person-centred hospital care for people with dementia. | To examine the feasibility and mechanism of the practice change. | Nurses (n=6) | Data collection: Focus group interviews  Data analysis: Thematic analysis | - Resources |  |
| Van Mierlo et al. (2015)  Netherlands  Nursing Home | Qualitative Study  A mental health care transfer intervention after the move of a person with dementia into a nursing home to promote continuity of care. | To evaluate the intervention and its implementation. | Community psychiatric nurses, professional nursing home carers, stakeholders (n=27) and family caregivers (n=5) | Data collection: Semi-structured interviews  Data analysis: Content analysis | - Financing issues - Organisational culture and vision - Resources - Staff turnover and fluctuations - Perceived value of the intervention - Collaboration with stakeholders - Team cultures - Knowledge, experience and skills of staff - Degree and clarity of responsibilities - Degree of familiarity with the intervention | - Organisation of the health insurance - Resources - Involvement of multiple disciplines and hierarchical levels - Team cultures - Motivation and openness of staff |
| Wils et al. (2017)  Belgium  Nursing Home | Before and after study  Educational program for nursing staff to improve advanced care planning, including training on theoretical aspects of advanced care planning and communication skills. | To explore the views of nursing home staff on advance care planning in patients with dementia. | Nurses (n=13) | Data collection: Semi-structured interviews  Data analysis: NI | - Resources - Nature and stage of dementia | - Management and leader support and engagement |

Abbreviations: DCM=Dementia Care Mapping; c-RCT=cluster-RCT; CCT=Controlled clinical trial; NI=No information available; RCT=Randomized controlled trial

1. Study design of the overall study (e.g. of the implementation or evaluation study wherein barriers and facilitators were obtained in an embedded sub-study or qualitative studies that stand on their own). [↑](#footnote-ref-1)
